# Deciphering the molecular landscape of rheumatoid arthritis offers new insights into the stratified treatment for the condition

**DOI:** 10.3389/fimmu.2024.1391848

**Published:** 2024-06-25

**Authors:** Min-Jing Chang, Qi-Fan Feng, Jia-Wei Hao, Ya-Jing Zhang, Rong Zhao, Nan Li, Yu-Hui Zhao, Zi-Yi Han, Pei-Feng He, Cai-Hong Wang

**Affiliations:** ^1^ Department of Rheumatology, Second Hospital of Shanxi Medical University, Taiyuan, China; ^2^ Shanxi Key Laboratory of Immunomicroecology, Taiyuan, China; ^3^ Shanxi Key Laboratory of Big Data for Clinical Decision, Shanxi Medical University, Taiyuan, China

**Keywords:** gene expression profiles, machine learning, rheumatoid arthritis, stratification, unsupervised clustering

## Abstract

**Background:**

For Rheumatoid Arthritis (RA), a long-term chronic illness, it is essential to identify and describe patient subtypes with comparable goal status and molecular biomarkers. This study aims to develop and validate a new subtyping scheme that integrates genome-scale transcriptomic profiles of RA peripheral blood genes, providing a fresh perspective for stratified treatments.

**Methods:**

We utilized independent microarray datasets of RA peripheral blood mononuclear cells (PBMCs). Up-regulated differentially expressed genes (DEGs) were subjected to functional enrichment analysis. Unsupervised cluster analysis was then employed to identify RA peripheral blood gene expression-driven subtypes. We defined three distinct clustering subtypes based on the identified 404 up-regulated DEGs.

**Results:**

Subtype A, named NE-driving, was enriched in pathways related to neutrophil activation and responses to bacteria. Subtype B, termed interferon-driving (IFN-driving), exhibited abundant B cells and showed increased expression of transcripts involved in IFN signaling and defense responses to viruses. In Subtype C, an enrichment of CD8+ T-cells was found, ultimately defining it as CD8+ T-cells-driving. The RA subtyping scheme was validated using the XGBoost machine learning algorithm. We also evaluated the therapeutic outcomes of biological disease-modifying anti-rheumatic drugs.

**Conclusions:**

The findings provide valuable insights for deep stratification, enabling the design of molecular diagnosis and serving as a reference for stratified therapy in RA patients in the future.

## 1 Introduction

Rheumatoid arthritis (RA) is a chronic autoimmune disease characterized by inflammatory polyarthritis, systemic inflammation, and autoantibody production. Uncontrolled RA results in progressive joint destruction and impaired functioning. Early and effective treatment using immunomodulatory medications is critical due to the potential for long-term chronic disease complications.

Nonsteroidal anti-inflammatory drugs, disease-modifying anti-rheumatic drugs (DMARDs), and glucocorticoids are commonly used conventional drugs for RA treatment. In recent years, DMARDs have assumed a more prominent role in managing RA and have been further categorized into conventional synthetic DMARDs (csDMARDs), biological DMARDs (bDMARDs), and target synthetic DMARDs according to the 2016 EULAR guideline (1). The 2021 ACR guidelines recommend starting treatment with csDMARDs in DMARD-naive patients. As second-line therapies, bDMARDs such as Rituximab and Infliximab are commonly utilized (2). Despite various available treatments, managing RA remains complex due to variations in practice based on the latest EULAR and ACR guidelines and challenges in predicting treatment outcomes and choosing the most effective medications (2). This underscores the necessity to identify and characterize patient subtypes that share similar goals and biological markers.

Currently, the widely utilized rapid assessment of disease activity in RA patients is the Disease Activity Score 28 (DAS28) (3, 4). However, DAS28 may not be suitable for all patients across different stages of RA and cannot precisely guide treatment decisions. Consequently, there is growing interest in developing more specific evaluation criteria and identifying predictors of response to biologics in RA. Recent studies have focused on the stratification of RA patients to predict prognosis and drug response more accurately. For example, a study by Lewis et al. utilized deep phenotypic profiling of RA synovial tissue, identifying transcriptional subtypes linked to three distinct cell-specific pathobiological modules (5). Their integration of ultrasonographic and radiographic data revealed that the plasma cell-infiltrated synovial module was associated with antibodies against citrullinated proteins (ACPA) positivity and a poorer prognosis in terms of radiographic damage at 12 months. Additionally, Jung et al. categorized RA synovial tissue gene expression into one inflammatory and two fibroblast subtypes, validating treatment responses in patients treated with a triple DMARD regimen (Methotrexate [MTX], Sulfasalazine, and Hydroxychloroquine) and Infliximab (6). These subgroups exhibited significant differences in ACPA positivity and treatment response. Moreover, Kraan et al. performed gene set enrichment analysis on peripheral blood data, identifying a high or low subtype based on the type I interferon (IFN) signature, and investigated the response to MTX treatment in this subtype (7). They found that IFN expression signature was a persistent trait in RA patients irrespective of MTX treatment. While these studies highlight the potential of stratifying RA patients for understanding disease pathogenesis and tailoring treatment more precisely, there remains a notable gap in research using large, publicly available peripheral blood databases, particularly concerning the response of stratified patients to bDMARDs. Therefore, it is imperative to leverage such publicly accessible data for RA stratification to refine individualized therapeutic strategies.

In the present study, we have constructed the largest genome-scale transcriptomic profiles of RA to identify patient subtypes sharing various pathways and signaling signatures. Additionally, we have evaluated the therapeutic outcomes of bDMARDs, providing a novel perspective for treating RA patients.

## 2 Methods

### 2.1 Data selection and processing

**Figure 1:**
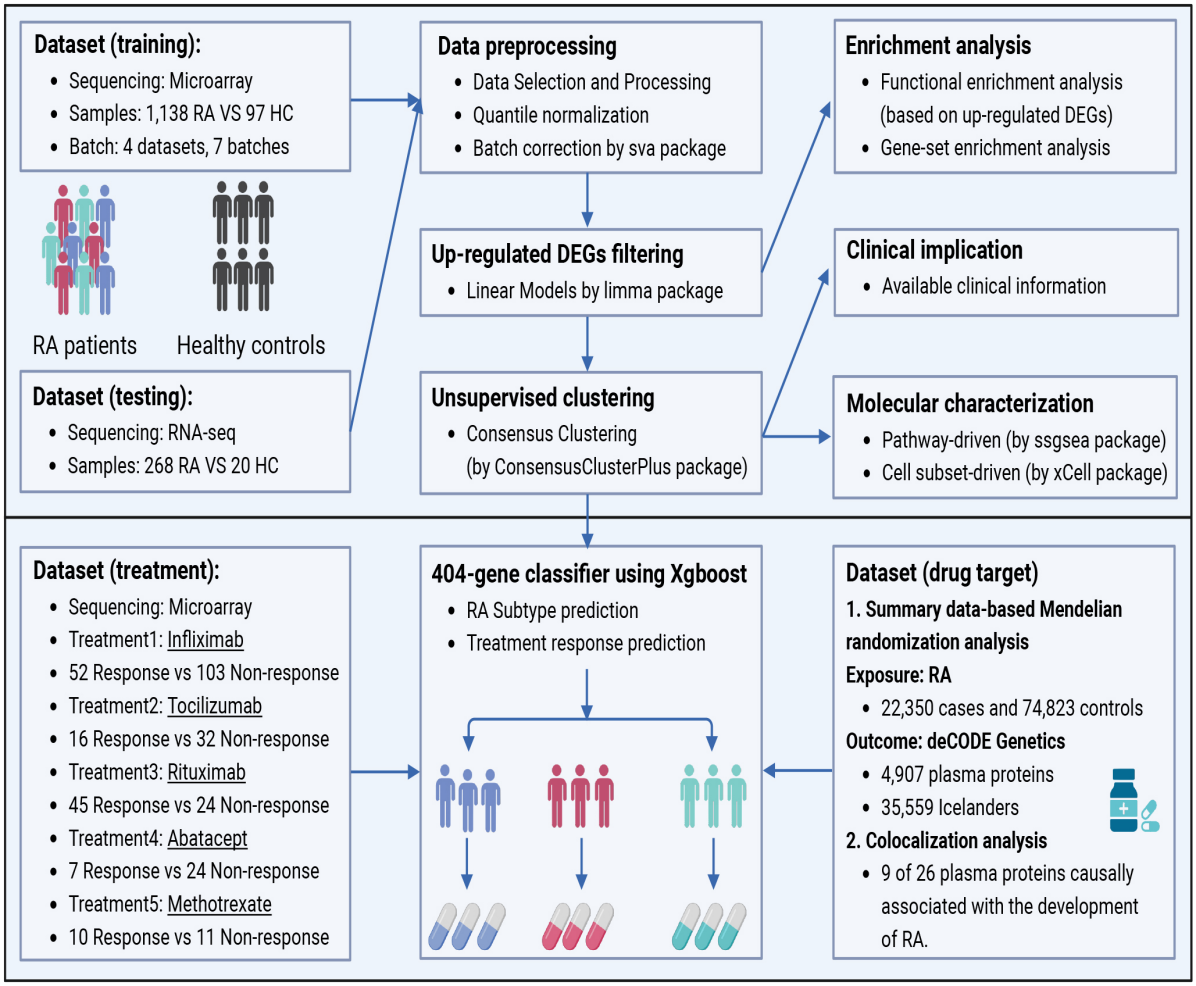
The workflow of data processing procedures in the study. Four microarray datasets containing 1,138 RA patients and three RNA-seq datasets including 268 RA patients were selected as training sets and test sets respectively from the public database. DEGs were filtered after the normalization, and unsupervised clustering was performed with enrichment analysis followed. Then, the XGBoost algorithm was contrived to predict the responses of stratified subtypes to commonly used five treatments. Finally, in search for more novel therapeutic targets of RA patients, drug prediction was carried out by utilizing SMR and the Open Targets platform. DEGs, differentially expressed genes; SMR, Summary data-based Mendelian randomization analysis.

The whole workflow of our study is presented in **Figure 1**. We selected four recently compiled, independent microarray datasets (GSE97810, GSE110169, GSE74143, GSE45291) (8–11) of RA peripheral blood mononuclear cells (PBMCs) from the Gene Expression Omnibus (GEO) database as the training sets. These datasets consisted of a total of 1,138 RA patients and 97 HCs. Additionally, three RNA sequencing (RNA-seq) datasets (GSE138746, GSE129705, GSE120178) (12–14) from the GEO database were collected as test sets, comprising a total of 268 RA patients and 20 HCs. Given that our study focused on investigating RA patients, more RA patients were enrolled to align well with real-world conditions and enhance its clinical relevance (15, 16).

We included several DMARDs in the analysis to study the association between RA peripheral blood subtypes and therapeutic response. Specifically, MTX (GSE93272), Infliximab (GSE93272, GSE78068 and GSE58795), Tocilizumab (GSE93272, and GSE78068), Abatacept (GSE78068), and Rituximab (GSE54629) (17–19). **Supplementary Table S1** provides more details on the study design and pre-processing of the microarray datasets.

The therapeutic response to conventional DMARDs such as MTX or biologic DMARDs (Infliximab, Tocilizumab [TCZ], Abatacept, and Rituximab) was evaluated by DAS28. Patients’ response was defined according to the EULAR response criteria (20, 21). For example, patients with a DAS28 score below 2.6 were considered responders, while those with higher scores were classified as non-responders.

To process the raw microarray data files from Affymetrix^®^, the robust multi-array average approach was used. It involved background correction, quantile normalization, and probe-set summarization using the ‘affy’ and ‘Simpleaffy’ R packages (22). The normalized matrix files for raw microarray data from Illumina were directly downloaded.

The expression profile of the RNA-seq data was converted to transcripts per kilobase millions format to allow for comparisons with the microarray datasets. To mitigate systematic, dataset-specific biases, the ‘ComBat’ function in the ‘sva’ R package was employed to adjust for residual technical batch effects resulting from integrating heterogeneous data (23). The ‘ComBat’ algorithm also showed its robustness when dealing with datasets from heterogeneous platforms, even the ones lacking HCs. Detailed validation information was presented in **Supplementary Tables S2**, **S3** and **Supplementary Figures S4**, **S5**.

To ensure quality assurance and assess distribution bias, principal component analysis (PCA) was conducted on the identical datasets before and after normalization and batch correction.

### 2.2 Differentially expressed genes obtaining and functional enrichment analysis

We performed differential gene expression analysis between RA patients and healthy controls using the “limma” R package, incorporating a linear model and a modified t-test (24). We adjusted the p-values using the false discovery rate (FDR) correction to account for multiple hypothesis testing. The Benjamin-Hochberg method was used to control the proportion of false-positive results (25). A threshold of adjusted p-value < 0.05 and log fold change (log FC) > 0.32 was set to determine the significance of DEGs.

To gain insights into the biological functions of the up-regulated DEGs, we conducted functional enrichment analysis using Metascape. This analysis included Gene Ontology (GO) annotation, Kyoto Encyclopedia of Genes and Genomes (KEGG) pathway enrichment, and Reactome. Enrichment terms with an adjusted p-value < 0.05 were considered significantly enriched (26).

### 2.3 Gene-set enrichment analysis

To investigate the potential biological processes or signaling pathways associated with the up-regulated DEGs between RA patients and healthy controls, we performed gene-set enrichment analysis (GSEA) using the GSEA software developed by UC San Diego and the Broad Institute (27–29). We obtained gene-set information on signaling pathways and biological processes from the KEGG and Reactome databases. Gene sets that were enriched among the up-regulated DEGs and had an FDR of less than 0.05 were identified.

### 2.4 Unsupervised clustering for gene expression-driven subtypes in RA patients

We utilized the R software “Consensus Cluster Plus” to perform hierarchical agglomerative clustering to subtype RA patients based on transcriptome signatures of PBMCs (30). The Partitioning Around Medoids algorithm, employing the Euclidean distance and Ward-D2 linkage, was applied for clustering the data. The core of Consensus clustering is to utilize resampling to generate subsamples from the original sample. These subsamples are then divided into a maximum of k groups and finally evaluated by analyzing the results obtained from multiple resampling returns. To ensure a robust stratification of subtypes and the consistency of the clustering results, we repeated the procedure across 1,000 reruns for k clusters. The number of clusters was determined using the cumulative distribution function (CDF). Unsupervised clustering results were also validated using PCA.

### 2.5 Molecular characterization by pathway and cell subset-driven enrichment analysis

In order to determine the activity levels of specific biological pathways within the three categorized subtypes, we employed single-sample gene-set enrichment analysis (ssGSEA) (31). This method utilizes an enrichment score to quantify the degree of enrichment in each sample for a given gene set within a dataset. We utilized publicly available resources, namely KEGG and Reactome databases, to identify RA-associated pathways. We also employed the “xCell” algorithm to calculate immune cell-type signature enrichment scores and determine cellular composition for the three subtypes (32). We utilized the Wilcoxon test to compare the enrichment scores, which represent pathway activity and cell-type signature, between any two of the three subtypes. Meanwhile, we employed the Kruskal-Wallis test for the comparison of all three subtypes. A p-value < 0.05 was considered statistically significant. Additionally, FDR correction was also applied to control the Type 1 error rate. Finally, the comparison of enrichment involving cell types and signaling pathways among subtypes would be considered significant at FDR < 0.05.

### 2.6 Development of an XGBoost classification model for subtype prediction

We constructed a decision tree using the XGBoost-tree approach in a multi-classification context with a softmax objective function to predict subtypes based on 404 gene characteristics (33). The receiver operating characteristic curve (ROC)’s area under the curve (AUC) was used to assess how well the prediction models performed. To train the classifier, 1,138 RA peripheral blood samples were divided into training (n = 799) and testing (n = 339) sets, with a respective ratio of 70% and 30%, utilizing the ‘caret’ R package. The subtype labels and expression values of the up-regulated DEGs were derived from the results of the unsupervised clustering approach. To mitigate overfitting, we used 10-fold cross-validation during the training process, and the fitted model was then utilized to assign subtypes to the testing sets using the 404-gene classifier.

### 2.7 SMR revealing the effects of plasma proteome on the risk of RA

To facilitate the discovery of RA drug targets, we used summary-data-based Mendelian randomization (SMR) and colocalization analysis for in-depth studies. Briefly, For the GWAS summary statistics for RA, we used the largest meta-analysis to date, comprising 22,350 cases and 74,823 controls of European origin (34). Quality control compared the RA GWAS data to the 1,000 Genomes Project Phase 3 European reference for the hg19 genome construction, excluded non-autosomal single nucleotide polymorphisms (SNPs), filtered out SNPs without rsID or with duplicated rsID and only kept biallelic SNPs with minor allele frequency (MAF) > 0.01. Summary-level statistics of genetic associations with levels of 1,881 plasma cis-protein quantitative trait loci (cis-pQTL) were extracted from 4,907 plasma proteins in 35,559 Icelanders (35). For each protein, we contained cis-acting SNPs (i.e. SNPs located within ±1 Mb of the gene body of the target gene). We used the cis-pQTLs as genetic instruments to evaluate the causal association between plasma proteins and the risk of RA.

SMR is a Mendelian randomization method that tests for shared causal variation between exposures and outcomes using summary-level data (36). In addition, we assessed whether the associations were in the presence of linkage disequilibrium using the heterogeneity in dependent instruments (HEIDI) test and removed single-nucleotide variants with p < 0.01. Based on the Benjamini–Hochberg method, FDR < 0.05 was considered statistically significant.

We performed further analyses using colocalization methods to determine whether there are potential shared causal variants between causal proteins and RA (37). A total of five posterior probabilities (PP) for mutually exclusive hypotheses were computationally generated (1). No genetic correlation for either trait (H0); (2) only trait 1 has a causal genetic variant (H1); (3) only trait 2 has a causal genetic variant (H2); (4) both traits have their own causal genetic variant, independent and different (H3); (5) both traits have the same shared causal genetic variation (H4). A colocalized locus was declared when the posterior probability of H4 (PP.H4) was greater than 0.7.

Finally, we searched the plasma proteins (PP.H4 > 0.7) using the Open Targets database to determine the potential druggability of the identified proteins (38).

### 2.8 Statistical analyses

For categorical variables, two groups were compared using the Wilcoxon test, and the Kruskal-Wallis test for comparisons involving more than two groups. To control the overall Type 1 error rate, FDR correction was also applied and FDR less than 0.05 was considered significant. As the variables for RA subtypes and clinical measures are numerical, we used the chi-square test or Fisher’s exact test to analyze the correlation between the two. Statistical significance was defined as p <0.05 for a two-tailed test. All statistical analyses were performed using R software (version 4.0.3).

## 3 Results

### 3.1 Screening and functional enrichment analysis of DEGs

**Figure 2:**
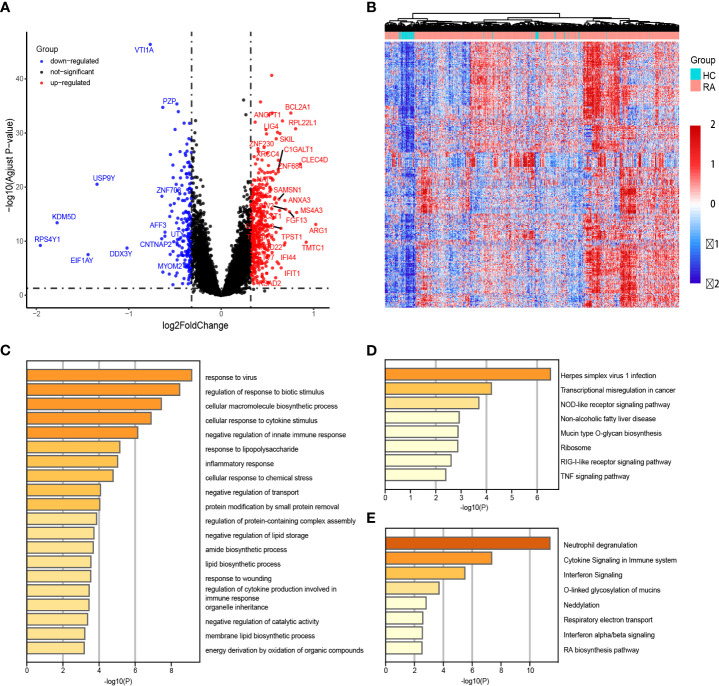
Identification of differentially expressed genes (DEGs) between patients with rheumatoid arthritis (RA) and healthy controls (HCs). **(A, B)** The heatmap and volcano plot of DEGs in RA patients versus HCs. **(C–E)** GO enrichment, KEGG and Reactome analyses of 163 up-regulated DEGs.

Upon comparing the gene expression profiles of peripheral blood samples from RA patients and HCs, we identified 404 up-regulated DEGs. These DEGs were visualized using volcanic and heat maps (**Figures 2A, B**). Functional enrichment analysis of the DEGs revealed gene ontology biological process terms that align with the current understanding of RA pathophysiology. Specifically, GO analysis indicated significant enrichment of biological processes such as response to viruses and bacteria, inflammatory response, organelle inheritance, and cellular macromolecule biosynthetic process (**Figure 2C**).

Additional KEGG analysis indicated that these elevated DEGs mostly enriched the TNF signaling, RIG-I-like receptor signaling, and NOD-like receptor signaling pathways (**Figure 2D**). Additionally, Reactome analysis demonstrated significant enrichment of the Interferon alpha/beta signaling pathways and Respiratory electron transport (**Figure 2E**).

### 3.2 Unsupervised cluster analysis identifies RA peripheral blood gene expression-driven subtypes

**Figure 3:**
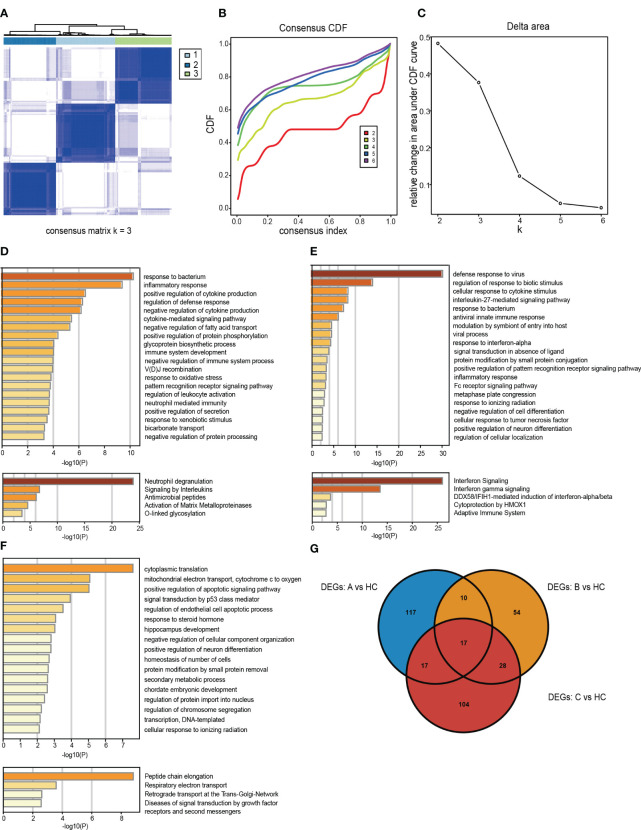
Identification and gene expression characterization of rheumatoid arthritis (RA) subtypes. **(A)** The consensus score matrix for RA samples when k = 3. A higher consensus score between the two samples indicated they were more likely to be assigned to the same cluster in different iterations. **(B)** Consensus clustering for the cumulative distribution function for k = 2–6. **(C)** Relative changes in the area under the cumulative distribution function curve for k = 2–6. **(D–F)** Molecular pattern distribution of three subtypes of RA in different biological processes and pathways. The top 20 most significantly enriched biological processes in each subtype of GO BP database and the top 5 most important signaling pathways in the Reactome database. **(G)** A Venn diagram showing up-regulated DEGs in subtype A, subtype B and subtype C compared with HCs.

We employed unsupervised cluster analysis to classify RA patients with different peripheral blood phenotypes based on the expression patterns of DEGs. This analysis was repeated 1,000 times to determine the optimal number of clusters, ranging from k = 2 to 6, using the CDF value and delta area as evaluation criteria. Our results indicated that k = 3 was the optimal number of subtypes (**Figures 3A–C**).

### 3.3 Molecular processes and biological functions of three subtypes in RA

To elucidate the potential pathological mechanisms underlying RA subtypes, we examined the molecular processes and their biological functions within each subtype (**Figures 3D–F**). By comparing, with HCs, the specific up-regulated DEGs signatures in the three subtypes, we identified 161 significantly up-regulated DEGs in subtype A, 109 in subtype B, and 166 in subtype C (**Figure 3G**). Next, using the GO Biological Process (GO-BP) and Reactome databases in Metascape, we explored the signaling pathways and most notably dysregulated biological processes in each subtype.

Subtype A was mainly enriched in biological processes related to response to bacteria, neutrophil-mediated immunity, and inflammatory response (**Figure 3D**). Subtype B exhibited significant activation of pathways involved in viral processes, including antiviral innate immune response, response to viruses, and interferon signaling (**Figure 3E**). Notably, subtype C was associated with protein synthesis processes, such as peptide chain lengthening, cytoplasmic translation, and transcription of DNA-templated genes (**Figure 3F**).

### 3.4 Molecular and cellular characterization of three RA subtypes

**Figure 4:**
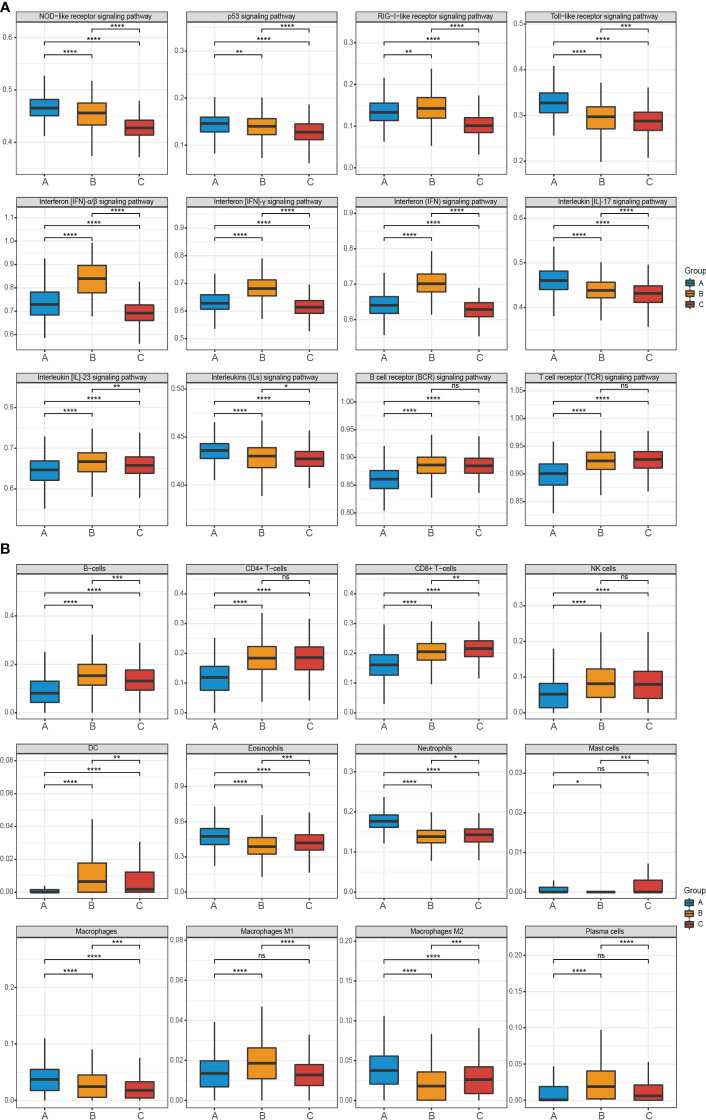
Pathway and cell subset-driven characterization in RA subtypes. **(A, B)** Enrichment scores for pathways and cell subsets for each RA subtype. Box plots for the enrichment scores of pathways and cell subsets for each RA subtype. Wilcoxon test was used to analyze the differences across three subtypes. ns, not significant; *P<0.05; **P<0.01; ***P<0.001; **** P<0.0001. FDR, false discovery rate.

The three clustered subtypes were labeled as subtype A (n = 399), subtype B (n = 356), and subtype C (n = 383). By comparing the enrichment scores of cell subsets and key RA-related pathways, we observed distinct immune-inflammatory characteristics among the three subtypes. The enrichment scores of 12 RA-related signaling pathways from literature, KEGG, and Reactome databases exhibited significant differences among the three subtypes after FDR correction (**Figure 4A**).

Specifically, subtype A was characterized by neutrophil activation, including pathways such as NOD-like receptor, Toll-like receptor, Interleukin (IL)-17, and various Interleukins (ILs) signaling pathways. On the other hand, subtype B exhibited prominent enrichment in IFN activation, including IFN-α/β and IFN-γ pathways. Subtype C did not show significant molecular characterization differences compared to the other two subtypes, however, subtype B and subtype C demonstrated greater significance than subtype A in the B cell receptor (BCR) and T cell receptor (TCR) signaling pathway.

Furthermore, using the xCell software and a machine learning framework, we estimated the enrichment of different cell types and validated differential activation across the three subtypes (**Figure 4B**). Subtype A displayed substantial infiltration of neutrophils, macrophages M2, and eosinophils. In contrast, B-cells, plasma cells, dendritic cells (DC), and macrophages M1 were more prominent in subtype B. Subtype C exhibited moderate enrichment in most cell types but had high expression of CD8+ T-cells.

### 3.5 Clinical implication of gene-driven subtypes in RA

**Figure 5:**
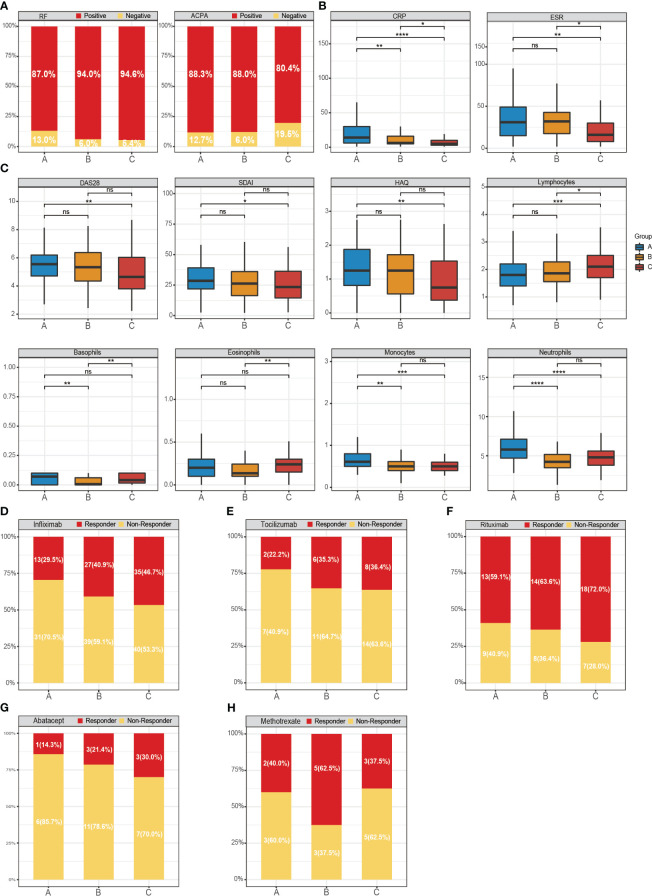
Distribution of gene-driven subtypes and multiple biologics treatments respond to the RA subtypes. **(A–C)** The variations across the three subtypes concerning autoantibodies, disease activity and four immune cells. The box plots show the disease activity scores of the three subtypes and the enrichment scores of immune cells. Responder: responded to the biologics; non-responder: did not respond to the biologics. **(D)** Responder/non-responder to infliximab: 29.5%/70.5% in subtype A, 40.9%/59.1% in subtype B and 46.7%/53.3% in subtype C. **(E)** Responder/non-responder to Tocilizumab: 22.2%/77.8% in subtype A, 35.3%/64.7% in subtype B and 36.4%/63.6% (0/5) in subtype C. **(F)** Responder/non-responder Rituximab: 59.1%/40.9% in subtype A, 63.6%/36.4% in subtype B and 72.0%/28.0% in subtype C. **(G)** Responder/non-responder to Abatacept 14.3%/85.7% in subtype A, 21.4%/78.6% in subtype B and 30.0%/70.0% in subtype C. **(H)** Responder/non-responder MTX: 40.0%/60.0% in subtype A, 62.5%/37.5% in subtype B and 37.5%/62.5% in subtype C. Wilcoxon test was used to analyze the differences across three subtypes. ns, not significant; *P<0.05; **P<0.01; ***P<0.001; **** P<0.0001.

To delve deeper into the association between molecular subtypes of RA and clinical attributes, we scrutinized the variations across the three subtypes concerning autoantibodies and disease activity. Autoantibodies serve as a distinctive feature of RA, notably rheumatoid factor (RF) and ACPA. Seropositive patients with the three subtypes of RA exhibited positivity for RF (A: 87.0%; B: 94.0%; C: 94.6%) and ACPA (A: 88.3%; B: 88.0%; C: 80.4%) (**Figure 5A**).

Moreover, we discovered that the levels of C-reactive protein (CRP) and erythrocyte sedimentation rate (ESR) in subtype A and subtype B were typically higher than in subtype C. Notably, CRP, commonly utilized as an indicator of systemic inflammation in RA, was abundantly expressed in subtype A (**Figure 5B**). Tools such as the Health Assessment Questionnaire (HAQ), DAS28, and Simplified Disease Activity Index (SDAI) are widely employed to evaluate disease activity. These tools corroborated that the disease activity index was notably higher in the subtype A of RA. However, these differences lacked statistical significance, possibly due to the constrained sample size.

Subsequently, we observed an elevated expression of neutrophils and lymphocytes in subtype A and subtype C, respectively, while the expression of basophils and eosinophils in subtype B showed a decrease (**Figure 5C**). These findings further corroborate the variances in clinical characteristics across the three subtypes.

### 3.6 Verification of classification results using RNA-seq datasets

We integrated three RNA-seq datasets from PBMCs, all comprehensively adjusted for batch effects and biases (**Supplementary Figures S1A–D**). To validate our classification results, these datasets encompassed 288 individuals, including 268 RA patients and 20 HCs. Using the gene expression profiles of 213 up-regulated DEGs, we segregated the patients into three subtypes: subtype A (n = 95), subtype B (n = 95), and subtype C (n = 80) (**Supplementary Figures S2A–E**).

We further examined the enrichment scores of RA-related pathways and cell subsets across the three subtypes (**Supplementary Figures S3A, B**). The consensus from our observations indicated that subtype A was principally enriched in neutrophil activation-related pathways and responses to bacteria. In contrast, subtype B exhibited an abundance of transcripts in IFN signaling and defense responses to viruses. Meanwhile, subtype C was notably associated with CD8+ T-cells.

### 3.7 Construction of a RA gene classifier and treatment responses of gene-driven subtypes

We devised a 404-gene classifier using an XGBoost machine learning algorithm to validate our RA subtyping scheme. We applied this classifier to a training set of 799 RA samples and a testing set of 339 RA samples. Our results attest to the practicality and robustness of this classifier, which successfully categorized the training set with an average area under the curve (AUC) value of 100%. Moreover, testing set validation achieved an accuracy of 89.68%, with an average AUC value of 90.99%. Hence, we concluded that the classifier serves as a viable and potent strategy for assessing RA subtypes in clinical trials.

Assessing the response to biological agents across different RA patients is crucial for elucidating the disease’s pathological specificity. Next, the 404-gene classifier was applied to predict the treatment response. We evaluated the response of Infliximab, TCZ, Rituximab, Abatacept, and MTX across the three RA subtypes (**Figures 5D–H**). Our findings indicated that subtype B (62.5%) exhibited a superior response to MTX compared to subtype A (40%) and C (37.5%). The three RA subtypes showed favorable responses to Rituximab, a B-lymphocyte-depleting agent, with subtype C achieving a response rate as high as 72%.

However, the response rates to Infliximab (a TNF inhibitor), TCZ (a humanized IL-6 receptor-inhibiting monoclonal antibody), and Abatacept (a T-cell co-stimulation modulator) across the different subtypes were comparatively low. In particular, subtype C consistently showed higher proportions of positive responses to Infliximab (46.7%), TCZ (36.4%), and Abatacept (30.0%) compared to subtypes A and B. In summary, the efficacy of certain targeted biological agents is intimately linked to RA patients with specific subtypes.

### 3.8 Causal proteins determined by SMR and colocalization analysis

**Figure 6:**
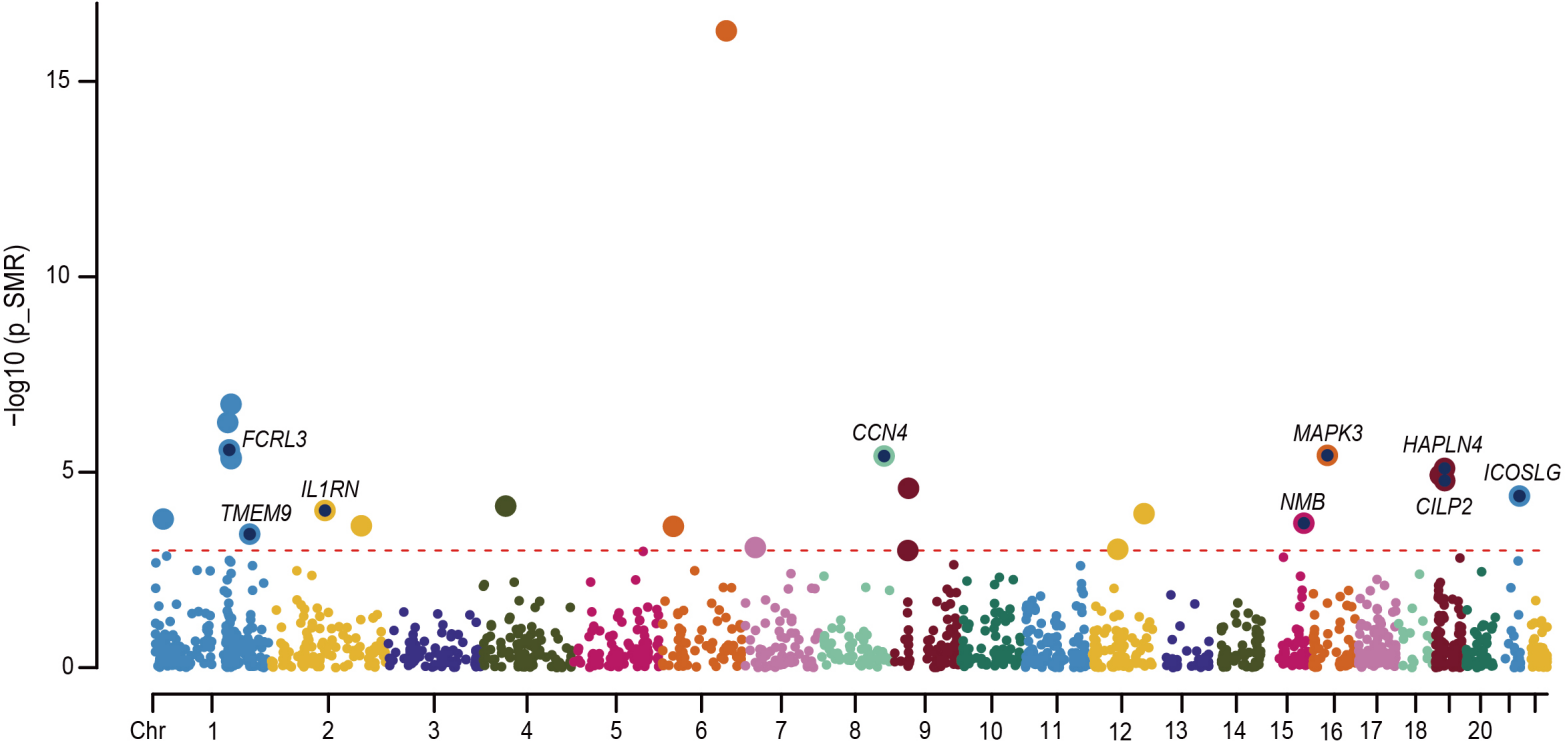
Manhattan plot for the 9 proteins identified in RA. Each point in the plot indicates a single association test between a plasma protein and RA as the -log10 (P) of a z-score test result which is ordered by genomic position on the x-axis and the association strength on the y-axis. The red horizontal line represents the significant threshold for the P value of FDR less than 0.05 under Bonferroni correction.

We performed an in-depth study of MR associations between 1,881 proteins with cis-pQTL and the risk of RA outcome using the SMR approach. We identified 197 unique proteins (SMR p < 0.05), of which 26 passed the HEIDI Test and FDR correction. Further colocalization analysis identified 9 proteins that may have significant roles in RA disease progression (PP.H4 > 0.7), including FCRL3, IL1RN, CCN4, NMB, MAPK3, HAPLN4, CILP2, ICOSLG, and TMEM9 (**Figure 6**). Among them, MAPK3 was identified as the highest-risk protein (FDR adjusted P = 2.56 × 10–4).

### 3.9 Druggability of the causal proteins

To further determine the potential new therapeutic targets of RA patients, we investigated the druggability of nine plasma proteins highlighted by colocalization analysis. Specifically, we identified six drugs targeting MAPK3 and ICOSLG proteins. For instance, AMG-557, an inducible co-stimulator (ICOS) ligand inhibitor, attenuates inflammation by inhibiting the accumulation of polyfunctional T helper 1 and T helper 17 cells. Ravoxertinib, Ulixertinib and MK-8353 serve as MAP kinase ERK1 inhibitors, and Temuterkib and KO-947, are ERK1/ERK2 inhibitors. They are all involved in the inflammatory response and tissue destruction in RA through mitogen-activated protein kinase (MAPK) and play a crucial role in the pathogenesis of RA. Therefore, whether the drugs that regulate these proteins can be reused for the treatment of RA requires further clinical experimental research.

## 4 Discussion

In this study, we explored differential expression patterns, significant pathways, and cellular components utilizing the most comprehensive microarray and RNA-seq datasets for RA to date. Employing an unsupervised cluster analysis, we identified three distinct subtype clusters. Subtype A was found to be enriched in neutrophil activation-related pathways and responses to bacteria. Subtype B, abundant in B cells, demonstrated an increased number of transcripts involved in IFN signaling and defense responses to viruses. Subtype C was discovered associating with CD8+T cells. These subtypes exhibited distinct clinical characteristics, including RF, ACPA positivity, and clinical assessments such as DAS28 scores.

Many preceding studies employing the stratification method have illustrated RA subtypes that can help predict potential prognoses for RA patients. Kraan et al. conducted a large-scale expression profiling by cDNA microarrays on peripheral blood, highlighting the identification of subtypes using complex IFN markers. They demonstrated that RA patients had much higher levels of IFN type I-regulated gene expression than healthy people. IFN-response genes showed increased expression in approximately half of the patients (IFN high patients). The IFN high group significantly varied from the IFN low group, according to pathway analysis, showing elevated pathways related to fatty acid metabolism, complement cascades, and coagulation (7). Our findings showed that subtype B aligns with the IFN cluster from the previous study. Furthermore, the enrichment of IFN signaling was also noted in other autoimmune disorders akin to subtype B of RA. For instance, Lanata et al. utilized an unsupervised clustering approach to stratify patients with systemic lupus erythematosus (SLE) and identified significant enrichment of genes associated with IFN signaling, antiviral responses, and inflammatory pathways (39). Mi et al. also identified two distinct IFN-1 subtypes in SLE patients. Surprisingly, they pointed out that IFN-1 might be a critical susceptibility factor for SS, potentially elucidating the pathogenesis of SLE patients who also develop SS (40). These findings strongly supported that identical signaling pathways exist in autoimmune diseases however they could also be clues for investigating the possible mechanism of the comorbidity of autoimmune diseases such as RA and SLE, proving the salience of investigating the stratification of patients.

Our study unveils that subtype A is associated with the inflammatory response, particularly the response to bacteria. The pathway of neutrophil degranulation, likely related to innate immunes such as the NOD-like receptor signaling pathway and ILs signaling pathway, is enriched in subtype A. We postulate that the delayed apoptosis, incited by NF-κB signaling activated by RA neutrophils, could exacerbate inflammation (41). With external pathogen irritation, the abnormal activation of the innate immune system and NOD-like receptor signaling may promote the secretion of ILs, which could trigger autoinflammatory and autoimmune responses. Subtype B contrasts with type A patients by displaying an abundance of interferon signaling, including both type-I and type-II interferon, CD+4 T cells, and B cells (42, 43). Naive CD4+ T cells become activated and differentiate into diverse T helper cell subsets that produce interferons in response to antigenic stimulation and cytokine signaling (44). IFN and IFN-related signaling pathways partly promote the inflammatory and adaptive response in RA patients. We also observed that both ACPA and RF show a relatively high positivity in subtype B patients. IL-21 produced by follicular helper CD4 T cells acts directly on B cells via their IL-21 receptors (IL21R), leading to the production of autoantibodies, including ACPA and RF (45–47). B cells are capable of producing both proinflammatory and anti-inflammatory cytokines, which might make the inflammatory state in peripheral blood for RA worse (48).

In addition to the above neutrophil activation and IFN signaling subtypes, CD+8 T cell factors also contribute to RA development. Due to ACPA+ RA being associated with major histocompatibility complex class II (MHC class II) HLA-DRB1 alleles, T cell studies in RA have focused primarily on CD4+ T cells. However, RA also exhibits genetic associations with alleles in the MHC class I HLA-B locus, highlighting the probable importance of CD8+ T cells (49, 50). It has been demonstrated that IL21R signaling in CD8+ T cells controls CD8+ T cell development and affects cellular metabolism, especially under conditions of persistent antigen presentation (46, 51, 52). In patients with subtype C, the abundant IL-21 produced by CD4+T cells plays a crucial role in RA development by acting on the IL-21R of CD8+T cells. Prior research and our results suggest that joint inflammation and destruction in RA are not exclusively antibody-driven and that IL-21/IL21R signaling may further drive autoimmune pathogenesis through autoreactive CD8+T cells (46).

To unravel and understand the disease heterogeneity of RA, we researched the response of biological agents to different RA subtypes by analyzing the notable therapeutic effect of Infliximab, TCZ, Rituximab, Abatacept, and MTX on patients of different subtypes. Treatment responses of gene-driven subtypes indicate that patients with specific subtypes may benefit more from some targeted biological agents than patients with other subtypes. Humby et al. has compared three non-tumor necrosis factor (TNF) α inhibitors drugs, including Rituximab, a B-lymphocyte depleter, Abatacept, which targets T-cell co-stimulation, and TCZ, an IL-6 receptor inhibitor. It was found that among adults with refractory rheumatoid arthritis, the results of Rituximab and TCZ were superior to those of Abatacept two years later, which aligns with our findings (53, 54). Furthermore, Rituximab, directed against CD20, affects the B cell population and reduces antibody production, therefore, demonstrating high therapeutic efficacy for the three subtypes. Infliximab exerts therapeutic effects on RA patients by inhibiting TNF-α binding to its target receptors and preventing the production of other proinflammatory cytokines, including IL and GCSF (55). Gerlach et al. observed that many CD8+T cells upregulate CX3CR1 upon pathogen challenge (56). Moreover, a study concluded that the CX3CL1-CX3CR1 system in patients with active RA might be sensitive to anti-tumor necrosis factor-alpha therapy and confirmed that CX3CL1 plays a critical role in the pathogenesis of RA, which may validate better therapeutic effects of Infliximab on C-type patients characterized by CD8+T cells compared to other types (57). MTX remains a cornerstone in treating rheumatoid arthritis and other rheumatic diseases. Previous studies showed that T cells isolated and activated ex vivo from RA patients treated with MTX have a diminished capacity to produce IFNγ, IL-4, IL-3, TNF, and granulocyte-macrophage colony-stimulating factor (58, 59). Over-activation of the interferon pathway, a characteristic pattern of mRNA expression, has been demonstrated in RA patients, and the same results have been observed in subtype B. Our work demonstrates great efficacy in some patient subtypes and indicates a potentially important result in efficacy that is now buried in unstratified analysis.

Furthermore, our SMR analysis based on cis-pQTL identified a total of six drugs including AMG-557, Ravoxertinib, Ulixertinib, MK-8353, Temuterkib and KO-947. Of these, AMG-557 as a class of autoimmune disease drugs has already completed the phase II clinical trial of Sjogren’s syndrome (NCT02334306) and phase I clinical trial of systemic lupus erythematosus (NCT02391259, NCT00774943 and NCT01683695) (60). Whereas Ravoxertinib, Ulixertinib, MK-8353, Temuterkib and KO-947 are currently used primarily to treat tumors. It is notable that both MAPK3 and ICOLSG, the protein targets of the above drugs, have now been shown to play a vital role in rheumatoid arthritis disease progression (61, 62). In the future, further in-depth studies are needed on the application of these drugs in RA subtyping therapy. Therefore, for this aspect, the pQTL analysis proposed in this study to identify drug targets as a more precise and personalized drug selection contributes to the new concept of precision medicine.

This study does have some limitations. First, additional meta-data would have been ideal, albeit this may be difficult given that this study was carried out in several clinical settings with various characteristics. Secondly, the Consensus clustering mainly relied on the subsampling which could lead to a reduction in data size and may result in potential clustering bias. Thirdly, the stability and potential changes in the proposed subtypes over time remain unknown due to the absence of longitudinal assessments within the descriptions of the subtypes in this review. Finally, a critical objective is to establish the correlation between clinically defined subtypes and biomarkers that reflect the underlying disease biology.

## 5 Conclusions

This endeavor holds significant potential for guiding the advancement of therapies tailored to specific subtypes. RA is a major medical challenge that requires more precise treatment. It is crucial to concentrate on the use of cutting-edge machine learning tools, as demonstrated in this study, in order to promote a better understanding of RA at a system level. We extensively analyzed the largest transcriptomic compendium for RA, utilizing the most comprehensive microarray and RNA-seq dataset available to date. These findings can serve as valuable guidance for developing molecular diagnostic approaches and as a future reference for tailored therapy in RA patients.

## Data availability statement

The original contributions presented in the study are included in the article/**Supplementary Material**. Further inquiries can be directed to the corresponding authors.

## Ethics statement

Ethical approval for this study was not required in accordance with the local legislation and institutional requirements. Written informed consent for participation in the original study was provided by the participants.

## Author contributions

MC: Conceptualization, Data curation, Formal analysis, Investigation, Methodology, Project administration, Software, Supervision, Validation, Visualization, Writing – original draft, Writing – review & editing. QF: Writing – original draft, Conceptualization. JH: Conceptualization, Data curation, Investigation, Methodology, Software, Validation, Writing – review & editing. YJZ: Conceptualization, Writing – original draft. RZ: Conceptualization, Investigation, Visualization, Writing – original draft. NL: Writing – original draft. YHZ: Data curation, Methodology, Writing – original draft. ZH: Investigation, Writing – original draft. PH: Funding acquisition, Project administration, Resources, Validation, Writing – review & editing. CW: Funding acquisition, Project administration, Resources, Validation, Writing – review & editing.
