# Supplementary material for: Deciphering the molecular landscape of rheumatoid arthritis offers new insights into the stratified treatment for the condition
Source: Front Immunol. 2024 Jun 25;15:1391848. doi: 10.3389/fimmu.2024.1391848 (PMC11232074; doi:10.3389/fimmu.2024.1391848)
Supplement: Supplementary file 1 [file DataSheet_1.docx]

Supplementary Material

# Supplementary Figures


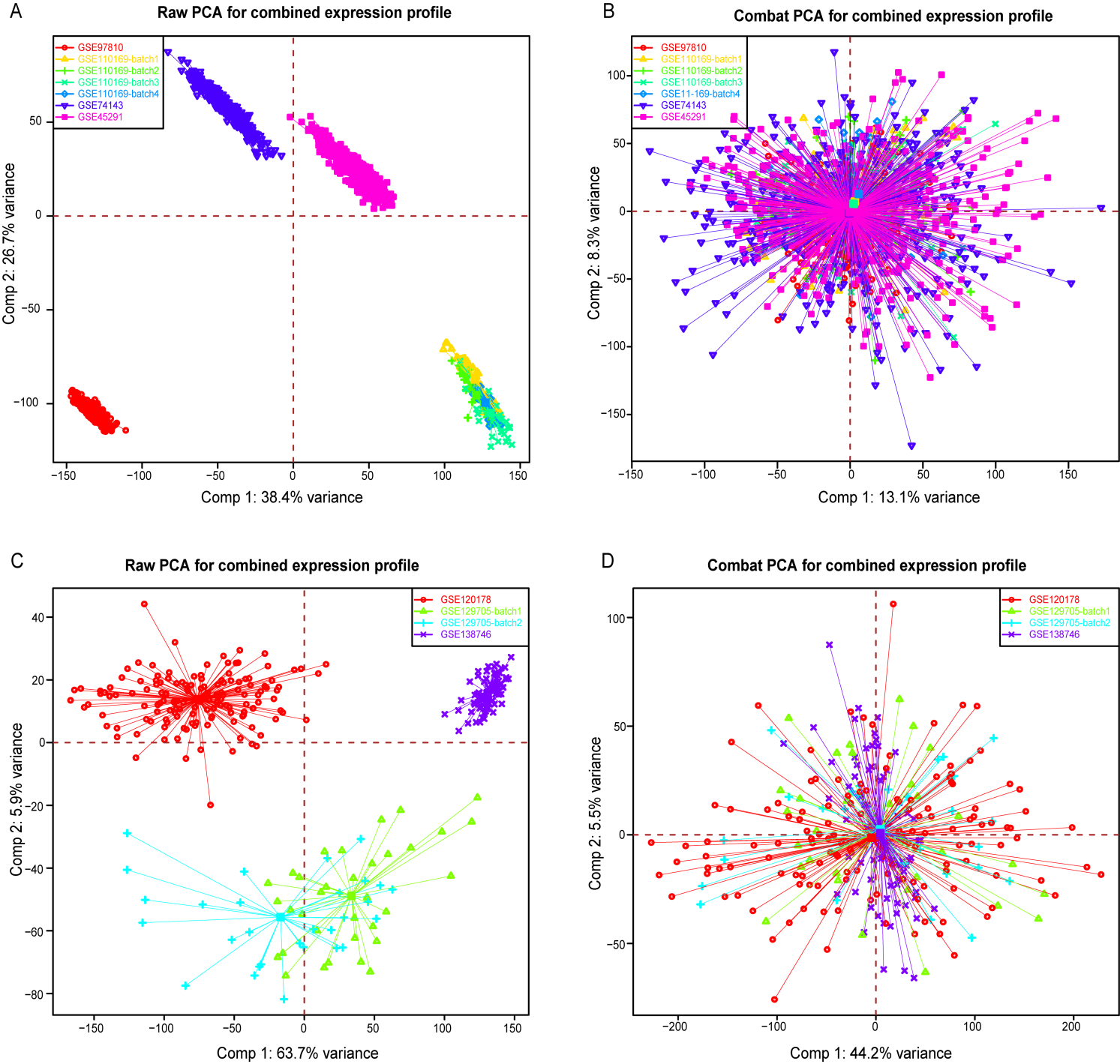


**Supplementary Figure 1.** **Principal component analysis plots with or without the elimination of batch effects.**

(A, C) Principal component analysis before batch effect adjustment for the training microarray datasets and testing RNA sequencing datasets. Samples from the different datasets cluster together. (B, D) Principal component analysis after batch effect adjustment for the training microarray datasets and testing RNA sequencing datasets. Samples from different datasets overlap.

**
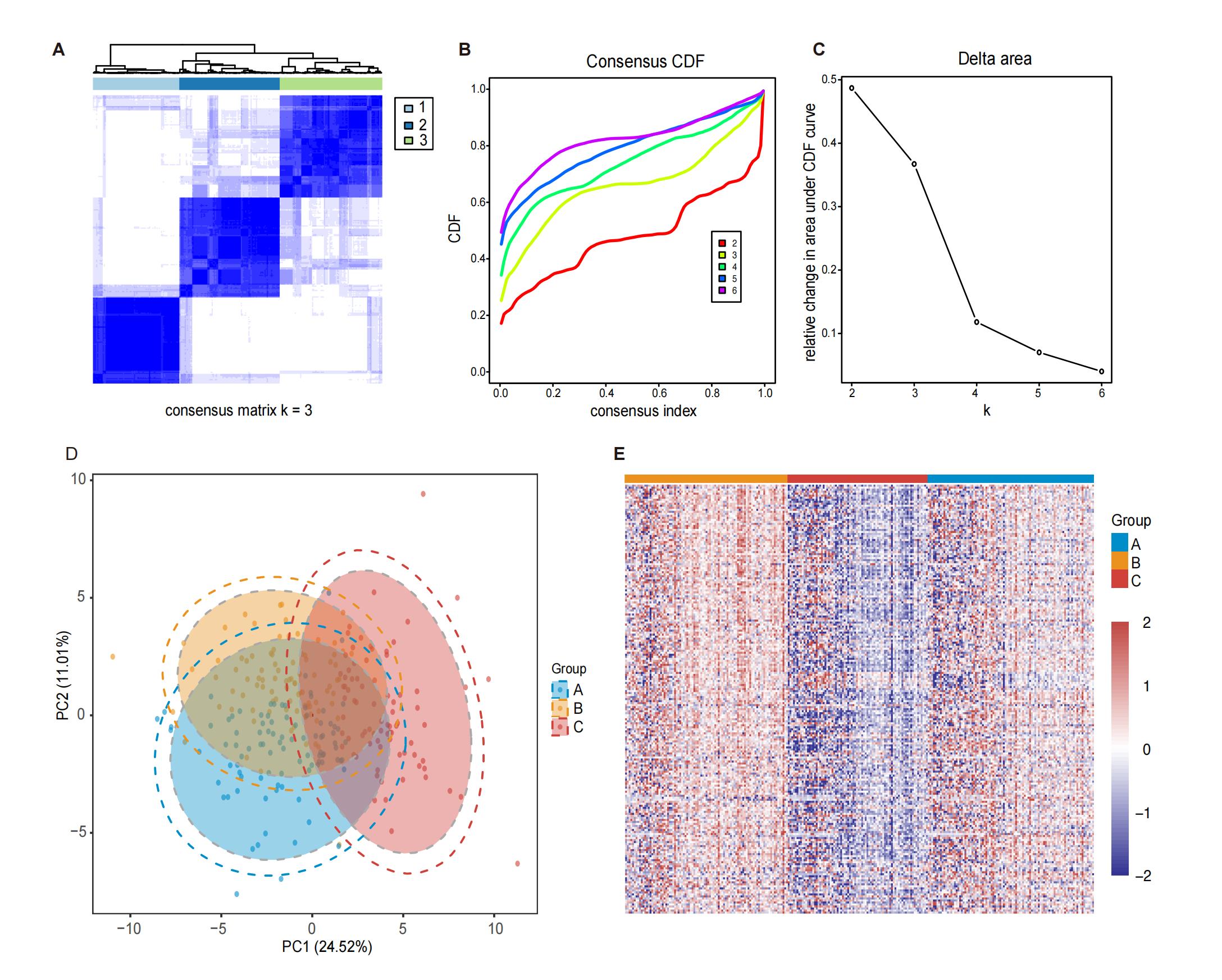
**

**Supplementary Figure 2. Identification of RA subtypes based on the up-regulated DEG signatures from the RNA sequencing datasets.**

The consensus score matrix for RA samples of RNA sequencing datasets when k = 3. A higher consensus score between two samples indicates that they are more likely to be assigned to the same cluster in different iterations. (B) Consensus clustering for the cumulative distribution function for k = 2-6. (C) Relative changes in the area under the cumulative distribution function curve for k=2-6. (D) Principal components analysis for the DEGs expression profiles showing the stability and reliability of the classification. (E) A heatmap of 100 RA patients with a red gradient illustrating the distribution of gene transcripts for three subtypes. In each column, patients are grouped based on cluster assignment.


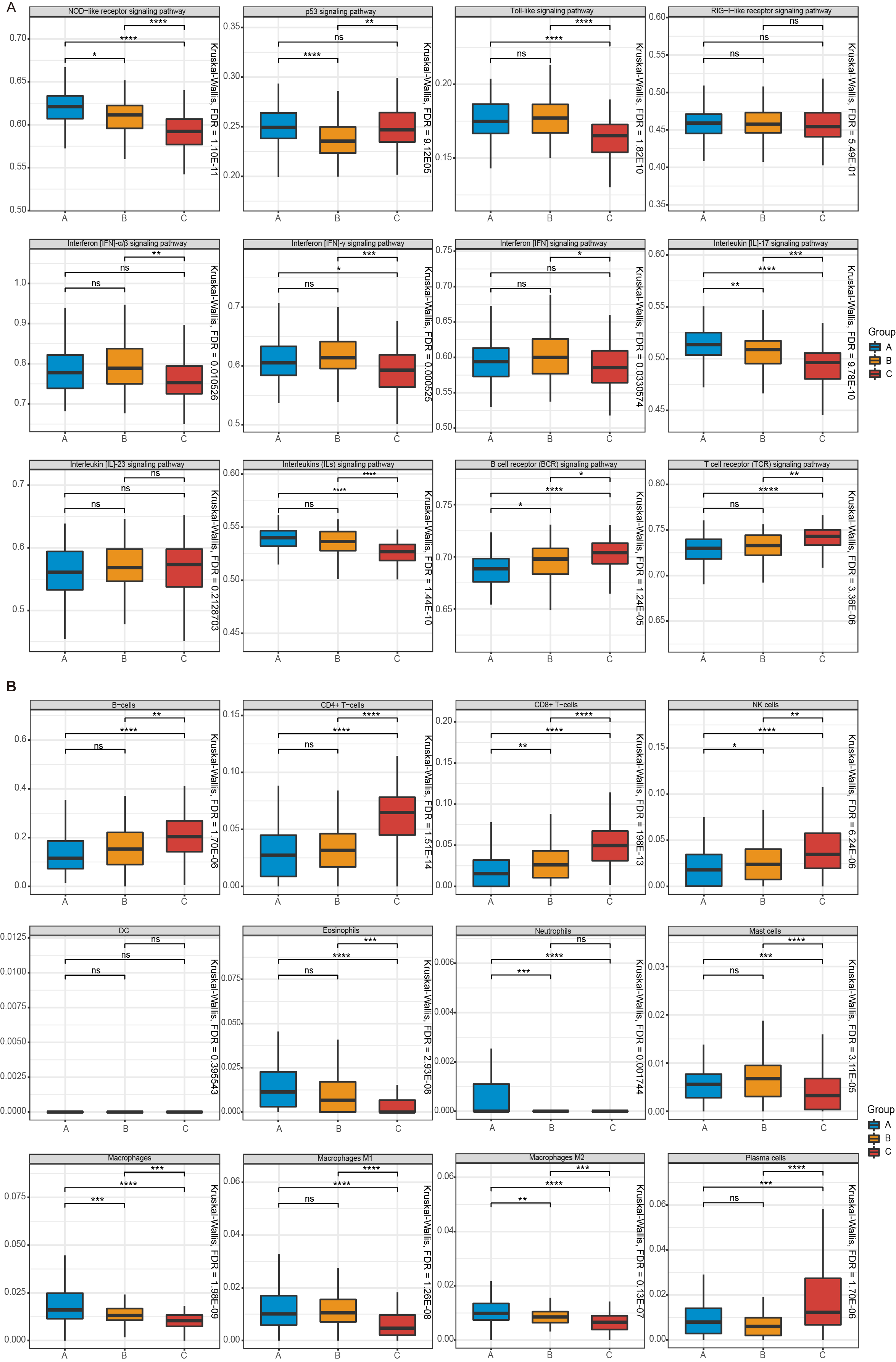


**Supplementary Figure 3. Pathway and cell subset-driven characterization in RNA sequencing datasets of RA subtypes.**

(A) Enrichment scores of pathways for each RA subtype. Box plots reveal pathway activation scores across the RA subtypes. (B) Cell subset enrichment scores according to RA subtypes. Differences across the three subtypes were analyzed using the Wilcoxon test. ns, not significant; *FDR < 0.05; ** FDR < 0.01; *** FDR < 0.001.

**Supplementary Figure 4. The heatmap of DEG results of the individual GSE110169 and GSE45291, RRA results, harmonized GSE110169 and GSE45291 results and harmonized four datasets.**Y-axis represents each value of log FC of individual GSE110169 and GSE45291, RRA results, harmonized GSE110169 and GSE45291 results and harmonized four datasets. The darker red represents higher log FC which is close to 1 while the darker green represents higher log FC which is close to -1.

**Supplementary Figure 5. The heatmap of DEG results of the individual GSE120178 and harmonized three datasets.**Y-axis represents each value of log FC of the individual GSE120178 and harmonized three datasets. The darker red represents higher log FC which is close to 1 while the darker green represents higher log FC which is close to -1.
