# Supplementary material for: Deciphering the molecular landscape of rheumatoid arthritis offers new insights into the stratified treatment for the condition
Source: Front Immunol. 2024 Jun 25;15:1391848. doi: 10.3389/fimmu.2024.1391848 (PMC11232074; doi:10.3389/fimmu.2024.1391848)
Supplement: Supplementary file 2 [file Table_1.docx]

Supplementary Material

# Supplementary Data

**Supplementary Table 1.** Rheumatoid arthritis **sample info.**

| **Data set** | **Subjects** | **Experiment type** | **Platforms** | **Collection site** | **Reference** |  |  |
| --- | --- | --- | --- | --- | --- | --- | --- |
| **microarray** | 1,138 RA vs 97 HC |  |  |  |  |  |  |
| Accession: GSE97810 | 184 RA vs 0 HC | Expression profiling by array | GPL10904 | Janssen R&D, United Kingdom | John C et al. | PMID: 35534493 | https://www.ncbi.nlm.nih.gov/geo/query/acc.cgi?acc=GSE97810 |
| Accession: GSE110169 | 84 RA vs 77 HC | Expression profiling by array | GPL13667 | Georgia Tech and Emory Univ, USA | Hu Y et al. | PMID: 29534336 | https://www.ncbi.nlm.nih.gov/geo/query/acc.cgi?acc=GSE110169 |
| Accession: GSE74143 | 377 RA vs 0 HC | Expression profiling by array | GPL13158 | Janssen R&D, USA | Walsh AM et al. | PMID: 27140173 | https://www.ncbi.nlm.nih.gov/geo/query/acc.cgi?acc=GSE74143 |
| Accession: GSE45291 | 493 RA vs 20 HC | Expression profiling by array | GPL13158 | BiogenIdec, USA | Petri M et al. | PMID: 25405351 | https://www.ncbi.nlm.nih.gov/geo/query/acc.cgi?acc=GSE45291 |
| **RNA sequencing** | 265 RA vs 20 HC |  |  |  |  |  |  |
| Accession: GSE138746 | 80 RA vs 0 HC | Expression profiling by high throughput sequencing | GPL20301 | University Medical Center Utrecht, Netherlands | Tao W et al. | PMID: 32909363 | https://www.ncbi.nlm.nih.gov/geo/query/acc.cgi?acc=GSE138746 |
| Accession: GSE129705 | 63 RA vs 0 HC | Expression profiling by high throughput sequencing | GPL16791 | Momenta Pharmaceuticals, Inc., USA | Farutin V et al. | PMID: 31647025 | https://www.ncbi.nlm.nih.gov/geo/query/acc.cgi?acc=GSE129705 |
| Accession: GSE120178 | 122 RA vs 20 HC | Expression profiling by high throughput sequencing | GPL16791 | Medimmune, Inc., USA | Guo X et al. | PMID: 30358109 | https://www.ncbi.nlm.nih.gov/geo/query/acc.cgi?acc=GSE120178 |
| **Treatment set (microarray)** |  |  |  |  |  |  |  |
| Accession: GSE93272 | Methotrexate  (10 Response vs 11 Non-response) | Expression profiling by array | GPL570 | Takeda Pharmaceutical Company Limited, Japan | Tasaki S et al. | PMID: 30013029 | https://www.ncbi.nlm.nih.gov/geo/query/acc.cgi?acc=GSE93272 |
| Accession: GSE93272 | Infliximab  (75 Response vs 110 Non-response) | Expression profiling by array | GPL570 | Takeda Pharmaceutical Company Limited, Japan | Tasaki S et al. | PMID: 30013029 | https://www.ncbi.nlm.nih.gov/geo/query/acc.cgi?acc=GSE93272 |
| Accession:  GSE78068 |  | Expression profiling by array | GPL6480 | DNA Chip Research Inc.,Japan | Nakamura S et al. | PMID: 27435242 | https://www.ncbi.nlm.nih.gov/geo/query/acc.cgi?acc=GSE78068 |
| Accession: GSE58795 |  | Expression profiling by array | GPL10379 | Merck Research Labs, USA | MacIsaac KD et al. | PMID: 25504080 | https://www.ncbi.nlm.nih.gov/geo/query/acc.cgi?acc=GSE58795 |
| Accession:  GSE93272 | Tocilizumab  (16 Response vs 32 Non-response) | Expression profiling by array | GPL570 | Takeda Pharmaceutical Company Limited, Japan | Tasaki S et al. | PMID: 30013029 | https://www.ncbi.nlm.nih.gov/geo/query/acc.cgi?acc=GSE93272 |
| Accession:  GSE78068 |  | Expression profiling by array | GPL6480 | DNA Chip Research Inc.,Japan | Nakamura S et al. | PMID: 27435242 | https://www.ncbi.nlm.nih.gov/geo/query/acc.cgi?acc=GSE78068 |
| Accession: GSE78068 | Abatacept  (7 Response vs 24 Non-response) | Expression profiling by array | GPL6480 | DNA Chip Research Inc.,Japan | Nakamura S et al. | PMID: 27435242 | https://www.ncbi.nlm.nih.gov/geo/query/acc.cgi?acc=GSE78068 |
| Accession: GSE54629 | Rituximab  (45 Response vs 24 Non-response) | Expression profiling by array | GPL6244 | Université Paris-Saclay, France | Sellam J et al. | NA | NA |
